# The eye as a complement dysregulation hotspot

**DOI:** 10.1007/s00281-017-0649-6

**Published:** 2017-09-25

**Authors:** Simon J. Clark, Paul N. Bishop

**Affiliations:** 1 Division of Evolution and Genomic Medicine, School of Biological Sciences, Faculty of Biology Medicine and Health, University of Manchester, Oxford Road, Manchester, M13 9PT UK; 2 Manchester Royal Eye Hospital, Manchester Academic Health Science Centre, Central Manchester University Hospitals NHS Foundation Trust, Manchester, UK

**Keywords:** Age-related macular degeneration, Complement dysregulation, Retinal disease, Inflammation, Immune tolerance, Clinical trials, Complement therapies

## Abstract

Complement turnover is tightly regulated throughout the human body in order to prevent over-activation and subsequent damage from inflammation. In the eye, low-level complement activation is maintained to provide immune tolerance in this immune privileged organ. Conversely, the complement system is suppressed in the cornea to protect it from continuous immunological insult. Over-activation of the complement cascade has been implicated in the disease progression of glaucoma and diabetic retinopathy and is now known to be a central driver in the pathogenesis of age-related macular degeneration (AMD). Indeed, it is with AMD where the most recent and exciting work has been carried out with complement-based therapies entering into clinical trials. However, the success of these trials will depend upon delivering the therapeutics to the correct anatomical sites within the eye, so a full understanding of how complement regulation is compartmentalized in the eye is required, a topic that will be highlighted in this review.

## Introduction

The role of the complement system in maintaining a healthy balance in immune homeostasis throughout the body is well recognized, and its role in the human eye is not less important. The importance of tight complement regulation and processing in the eye is highlighted in a number of eye disorders where control breaks down, with age-related macular degeneration (AMD) being a prime example. Recent studies that have linked genes of the complement system with risk of developing AMD [1, 2] and this has spurred an increased interest in understanding complement-mediated ocular immunity and the development of new complement-modifying therapeutics.

### Complement homeostasis in the healthy eye

Continuous low-level turnover of complement within the eye has been recognized for many years [3] and is known to be a major contributor to the immune privilege status of the eye [4]; for example, the major component of immune privilege, termed anterior chamber-associated immune deviation (ACAID), has been shown to be complement-dependent [5]. The binding of iC3b to the CR3 receptor on antigen-presenting cells (APCs) induces the production of TGF-β2 and IL-10, which are essential for the induction of tolerance [5]; this phenomenon has also been observed in other tissues [6, 7]. So, despite being a major opsonin and mediator of inflammation, iC3b deposition has important protective roles inside the eye.

Complement has a well-established role in the maintenance of a healthy cornea [8]. Although membrane-bound complement regulators such as CD46, CD55, and CD59 are expressed throughout the various layers of the cornea, there is a disproportionately high level of expression in the corneal epithelium [9]. This is important as the corneal surface is subject to constant exposure to various pathogens including bacteria such as *Pseudomonas aeruginosa* [10], and consequently, continuous complement activation. Indeed, some bacteria produce phospholipases and other enzymes that can remove the GPI-anchored CD55 and CD59 from the corneal epithelial surface [11], potentially leaving the cornea susceptible to dysregulated complement activation that exacerbates bacterial keratitis (inflammation of the cornea) and can lead to loss of vision [10].

### Ocular disease and the complement system

Clearly, given that the fine balance between complement activation and suppression is so important in maintaining a healthy environment within the eye, when it is perturbed, deleterious effects can occur leading to, or contributing towards, disease. Several eye diseases involve the complement cascade in their pathogenesis including the following.

#### Glaucoma

The term “glaucoma” refers to a family of ocular diseases that are characterized by damage to the optic nerve and subsequent visual loss. The global prevalence of glaucoma in populations aged between 40 and 80 years is 3.5% [12], and it is predicted that as the global population ages, 76 million people will suffer from the disease by 2020, rising to 111 million in 2040 [12]. While the exact underlying causes of glaucoma remains unclear, a central risk factor is the intraocular pressure (IOP) being sufficiently high to damage the optic nerve. Although increased IOP is an important sign of glaucoma, it is not a definitive marker as patients can develop glaucoma with a “normal” IOP [13]. The cells that are primarily damaged in glaucoma are the retinal ganglion cells (RGCs) as they form the axons of the optic nerve. The first implication of complement involvement in glaucoma came from studies performed in 2003 using gene microarray analysis on a disease model of glaucoma in cynomolgus monkeys, where retinal C4 and properdin gene transcription was elevated in both mild and severe glaucoma and C3 and C1q gene transcription up-regulated in severe glaucoma [14]. Subsequently, it was shown that C1q was specifically up-regulated in murine, primate, and human retina during early-stage disease, prior to any detectable retinal damage, and continued to increase in line with the progression of glaucomatous neurodegeneration [15]. These findings have been replicated in other human glaucoma samples as well as rat models of induced ocular hypertension [16], where the latter also showed disease-associated up-regulation of C3 in RGCs [16]. Additionally, inhibition of the classical complement pathway in both genetic (DBA/2J mouse) and inducible (rat microbead) models of glaucoma resulted in reduced RGC loss, thus providing a clear link between classical complement activation and accelerated RGC damage in disease [17]. Despite these associations, the exact mechanism by which complement contributes to the development of pathology in glaucoma remains unclear and is the subject of a number of on-going studies.

#### Diabetic retinopathy

Diabetic retinopathy is a leading cause of preventable blindness around the world; about one-third of the diabetic population suffers from some stage of retinopathy [18]. Clearly, diabetes itself is not a complement-mediated disease, but some data do exist that imply a link between progression of diabetic retinopathy and complement dysregulation. For example, the deposition of C5b-9 complexes has been observed within the retinal blood vessel walls of both diabetic rats and humans [19]. In addition, reduced levels of the two membrane-bound complement regulators linked to plasma membranes via glycosylphosphatidylinositol anchors were observed in diabetic retinas, i.e., CD55 and CD59, but not of CD46 which is transmembrane [20]. Furthermore, there is evidence that CD59 can be inactivated by non-enzymatic glycation [21, 22]. However, it still remains unclear whether these observed changes in complement activation and regulation actually contribute towards disease progression or are simply a consequence thereof. There have been recent genetic association studies linking the *C5* gene with diabetic retinopathy. One study found the intronic SNP rs2269067 in the *C5* gene to be associated with proliferative diabetic retinopathy in the Chinese Han population, and this SNP was shown to be associated with increased C5 mRNA expression [20]: the study also noted an increase in the production if IL-6 associated with the rs2269067 SNP. Another study found a weak association between the coding SNP rs17611 (which results in the V802I polymorphism in the C5 protein previously associated with rheumatoid arthritis [23]) and diabetic retinopathy in type 2 diabetes [24]. Given the data linking diabetic retinopathy to complement over-activation, it has been suggested that anti-complement therapeutics have potential for treating diabetic retinopathy [22].

#### Autoimmune uveitis

Uveitis is a term describing a group of inflammatory conditions that damage the eye internally. Depending on which part of the eye is affected, uveitis can be anatomically classified as anterior, intermediate, posterior, or diffuse (panuveitis). Autoimmunity is an important cause of uveitis, but other causes include infections, tumors, trauma, and toxins. While the role of the adaptive immune response in uveitis has been well studied for a number of years [25], a potential role of complement activation has only recently been identified [26, 27]. For example, complement activation in the eye appears critical for driving the local production of cytokines (IFN-γ and IL-10), chemokines (IP-10), and adhesion molecules (ICAM-1 and LECAM-1) in a Lewis rat experimental model of anterior uveitis [26]; furthermore, depletion of complement in this model resulted in inhibition of disease. Despite the resulting argument for targeting the complement system with therapeutics for some forms of uveitis, more research is required into disease mechanisms before embarking on such interventions.

#### Age-related macular degeneration

**Fig. 1:**
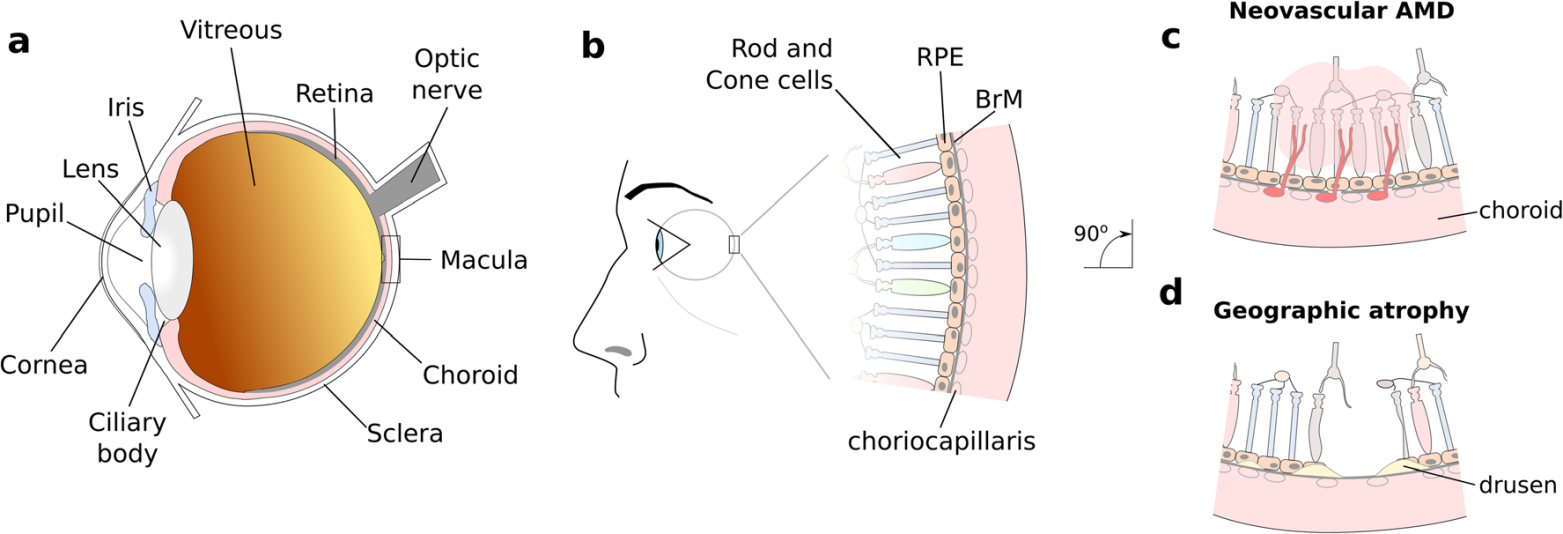
Schematic showing the layout of tissue in the macula of the human eye. **a** Diagram highlighting regions of the human eye. **b** AMD manifests by the progressive destruction of the macula, the central part of the retina at the back of the eye. Early stage disease is characterized by the formation of lesions (termed drusen) in Bruch’s membrane (BrM). The disease then progresses onto late-stage disease, which is divided into either neovascular “wet” AMD **(c)** or geographic atrophy **(d)**, both of which result in loss of visual acuity

Dysregulation of the tightly controlled complement cascade has many consequences for disease progression throughout the body and AMD provides an excellent example of how aberrant complement turnover can drive forward disease pathogenesis. AMD results in progressive destruction of the macula, the central part of the retina which is responsible for high resolution vision (see Fig. 1). AMD is a highly prevalent disease; it is currently responsible for 8.7% of all blind registrations globally, and it is projected that 196 million people worldwide will suffer from some form of AMD by 2020 [28]. In early stages of the disease, morphological changes in the macula occur (see Fig. 1b), including loss of blood vessels in the choriocapillaris [29], a layer of fenestrated capillaries immediately underlying Bruch’s membrane (BrM). Complement activation and turnover has been demonstrated in the choriocapillaris layer, and increased levels occur in AMD and may precede the condition [29–31].

BrM is a five-layered sheet of extracellular matrix [32] that separates the retinal pigment epithelium (RPE) from the choroid (Fig. 1b), and it is within the BrM that the hallmark lesions of early AMD form, termed drusen. Drusen are deposits containing lipids and proteins and include a swathe of complement activation products [33, 34]. Drusen disrupt the flow of nutrients and cellular waste products across BrM, and this can eventually lead to cell death. This cell death typically occurs in patches that clinically are referred to as geographic atrophy; this is a form of late AMD, i.e., AMD associated with visual loss. The other form of late AMD is neovascular AMD, where pathological blood vessels grow from the choroid into the retina (i.e., choroidal neovascularization). This neovascularization is largely driven by excessive vascular endothelial growth factor (VEGF) production, especially by the RPE, and is treated by injecting anti-VEGF agents into the eye thereby limiting visual damage. Geographic atrophy remains untreatable.

Although lifestyle factors such as diet and smoking are associated with increased disease risk, AMD is largely genetically driven and several of the genetic variations associated with modified AMD risk reside in or near genes of the complement cascade (reviewed in [35]). Single nucleotide polymorphisms (SNPs), and in some cases rare variants, in the *CFH*, *CFI*, *CFB*, *C3*, and *C9* genes, as well as the complete deletion of the complement factor H-related (*CFHR*) genes *CFHR-1* and *CFHR-3*, are associated with modifying an individual’s risk of developing AMD [35, 36]. Various studies have identified the chromosome 1 locus at 1q32 (that contains the *CFH* and five *CFHR* genes) as being one of two major risk loci for AMD [37] (the other is on chromosome 10 (10q31) around the *ARMS2/HTRA1* genes [38]). Rare variants strongly associated with AMD have been found in the CFH, CFI, and *C9* genes [39, 40]. The genetic associations, coupled with the observed accumulation of complement proteins and markers of complement activation in the macula in AMD [31, 33, 41], have highlighted the role of complement activation (especially via the alternative pathway), and this is now a major focus for the development of therapeutics [42].

One of the major SNPs associated with increased risk of developing AMD is in the *CFH* gene and encodes the Y402H polymorphism. This polymorphism is in the main fluid phase regulator of complement, factor H (FH) [43–47], and its alternative splice variant factor H-like protein 1 (FHL-1) [43, 48]. In addition to acting in the fluid phase, FH and FHL-1 can inhibit complement on cell surfaces and extracellular matrix by acting as a co-factor for Factor I and through their decay accelerating activity against C3-convertase. Around 30% of individuals of white European heritage have at least one copy of the risk (402H) variant of this polymorphism and have an ~ 3-fold increase in AMD risk compared to individuals with 402YY, and 5–6% of Europeans are 402HH with an ~ 5-fold increase in AMD risk [37].

### Systemic versus local complement activation in AMD

One unresolved aspect of complement turnover in relation to AMD is whether local or systemic complement activation, or both, contributes to disease pathogenesis. Originally, significantly elevated levels of the inactive form of C3a (C3a-desArg) were measured in plasma from AMD patients [49]. A subsequent study replicated this and also found that levels of Ba and C3d were particularly elevated [50] thereby providing evidence that chronic systemic complement activation is associated with AMD. However, there are inconsistencies between studies. For example, complement factor D (FD) levels in plasma were decreased in AMD samples in one study [51], but elevated in another [52], and the association of elevated plasma levels of the terminal complement complex (TCC) with AMD have been similarly disputed [50, 53–55]. A trial using systemically delivered eculizumab, an antibody therapeutic that targets C5, failed to slow the progression of geographic atrophy [56], suggesting that systemic inhibition of the terminal complement pathway is an ineffective approach for treating late AMD.

Despite some evidence of associations between systemic complement protein levels and AMD, and genetic associations linking complement with AMD, the link between AMD-associated SNPs and systemic complement turnover is much less clear. AMD-associated SNPs in the *C3* and *CFH* genes (rs2230199 and rs800292, respectively) were reported to alter complement turnover as measured by the C3d/C3 ratio [57]. However, while one study replicated this finding for the *C3* rs2230199 SNP, also reporting increased C5a levels [54], another study failed to replicate the finding [58]. A single study reported an association between the common disease risk-associated Y402H polymorphism in *CFH* and systemic complement turnover [53], but several other studies did not reproduce this finding [49–51, 54, 57, 58]. A study investigating patients who had liver transplants found that AMD was not associated with liver FH production or systemic complement activation in liver transplant patients [59]. This suggests that local intraocular complement production/activity in the eye is of greater importance in determining AMD risk. Furthermore, individuals homozygous for the *CFH* 402H risk variant have increased deposition of complement activation markers in the choriocapillaris and around BrM compared to those with a single or no risk alleles [31, 60], supporting the concept that complement activation within the eye is important in AMD.

#### Bruch’s membrane

FH and FHL-1 are the only components of the alternative pathway capable of suppressing complement activation on extracellular matrix. Immunolocalization studies have demonstrated FHL-1 as the major form in BrM and the intercapillary septa of the choriocapillaris, while small amounts of FH are deposited in the inner surface of Bruch’s membrane (at the interface with the RPE) and around the vessels of the choriocapillaris [61]. FH and FHL-1 are synthesized by the RPE as well as potentially being supplied to this region by the blood in the choriocapillaris. However, the large (155 kDa), glycosylated FH protein cannot diffuse through BrM, while the 50 kDa, non-glycosylated FHL-1 can [61], thereby explaining why FHL-1 is the predominant form in these extracellular matrices.

Once in BrM or the intercapillary septa, FH/FHL-1 binding to these extracellular matrices is mediated by sulfated sugars including the glycosaminoglycans (GAGs), heparan sulfate (HS), and dermatan sulfate (DS) [62–64]. The family of GAG sequences found in BrM and the intercapillary septa of the choriocapillaris have a high specificity for the GAG-binding site in CCP7 of FH/FHL-1 rather than the other major GAG-binding site of FH in CCPs19–20 (the truncated FHL-1 only contains CCPs1–7) [65–68]. As FHL-1 only has the one GAG-mediated anchoring site in its CCP7 domain, it is this that anchors it to BrM and the intercapillary septa. This GAG-binding site is affected by the Y402H AMD-associated polymorphism, with the 402H form binding less well to GAGs in these structures. This provides a potential explanation as to why the Y402H polymorphism is associated with AMD, with the 402H form of FHL-1 (and FH) binding less well to the extracellular matrix leading to complement activation on BrM and in the intercapillary septa and this in turn resulting in inflammation. An age-related decrease in the amount of HS and DS has been detected in BrM, possibly providing an explanation for the age-related nature of AMD [68, 69]. The Y402H polymorphism is not associated with kidney disease, whereas the CCP19–20 domain of FH is known to be the main GAG-mediated anchoring site in the extracellular matrix of the kidney, and mutations in this region are found in renal disease [65].

While the above findings suggest a role for CCP7 binding to extracellular matrix in AMD pathogenesis, FH influences AMD risk through other mechanisms. A rare mutation (R1210C) in the C-terminal (CCP19–20) region of FH is strongly associated with AMD [70]. FH protein carrying this mutation is found in the plasma covalently bound to albumin [71], and it should be noted that it is so rare that only individuals heterozygous for the mutation have been found; in these, AMD risk in these individuals is also associated with the presence of the 402H variant in FH [70]. Binding to albumin likely confers a reduced capacity for binding C3b, perturbation of mobility/diffusion, or perhaps both [71].

#### Choriocapillaris

As discussed above, there is evidence of increased TCC and C3 deposition in the intercapillary septa of the choriocapillaris in patients who are homozygous risk for the common risk allele on chromosome 1. There is also increased deposition of C-reactive protein (CRP) deposition in the choriocapillaris in AMD [60, 72]. This acute-phase inflammatory protein, itself an activator of complement, circulates as a non-inflammatory pentameric protein (pCRP), which dissociates into a monomeric form (mCRP) upon surface binding [73]. While FH cannot bind pCRP, it can bind mCRP and dampen complement activation [74]. The Y402H polymorphism associated with AMD significantly reduces the capacity of FH to bind mCRP [74, 75], and this may result in over-activation of complement in the choriocapillaris.

It has been suggested that the large drusen that precede geographic atrophy and the associated pigmentary changes in the RPE cells of early AMD indicate that geographic atrophy results firstly from dysfunction of the RPE cells with secondary effects upon the choroid [76]. By contrast, as previously discussed, Whitmore and colleagues have noted changes in the choriocapillaris, including the deposition of the terminal MAC, preceding all forms of late-stage AMD, and argue that this is the primary event with RPE changes being secondary [29]. Certainly, almost all evidence presented in the literature to date shows complement activation within the choriocapillaris and intercapillary septa. This would imply that a genetic predisposition, conferred by alterations in complement genes, is tolerated until local morphological and biochemical changes in the macular region create an environment where spontaneous complement activation can no longer be regulated, and this ultimately leads to sight loss from AMD.

#### BrM creates two semi-independent compartments with respect to complement activation and regulation

**Fig. 2:**
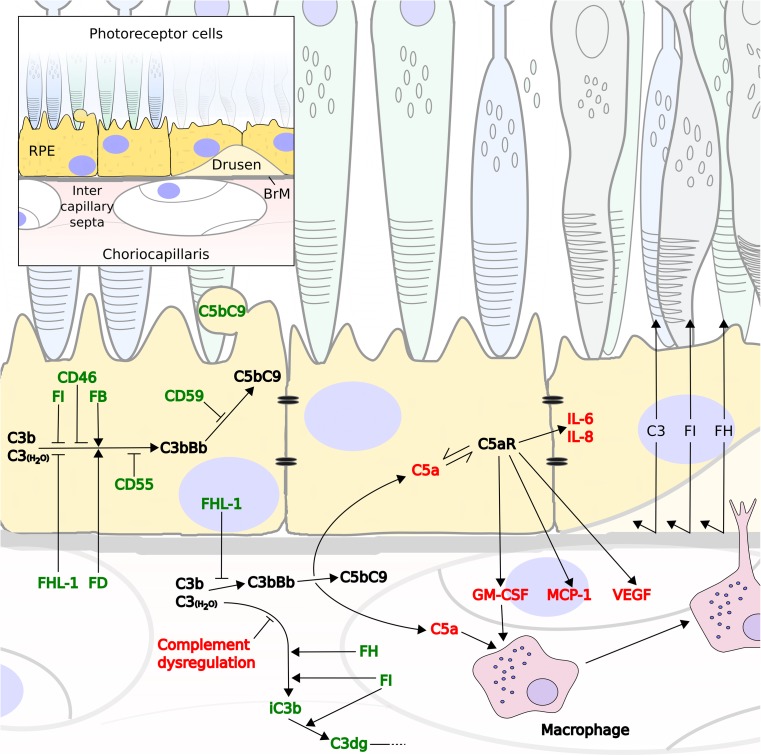
Normal complement turnover in the retinal space may be aggravated by excessive complement activation in the choroid. Flow diagram demonstrating that C3b (or C3(H_2_O)) deposited in either the RPE cell layer or intercapillary septa is broken down in order to prevent the runaway activation of the complement cascade (green). Regulation includes the blebbing off of RPE membrane where C5b-9 complexes have been deposited. However, failure of complement regulation in the ECM of the choriocapillaris drives forward complement activation at this site that can illicit responses elsewhere (red). Positive complement regulators could compete with FH for the binding of C3b, thus preventing its breakdown to iC3b and promotes instead the drive towards C3 convertase formation (C3bBb). Increased complement turnover generates complement deposition in the choriocapillaris and the anaphylatoxin C5a that can diffuse across BrM and stimulate RPE cells via their C5a receptor (C5aR). This leads to an increase in IL-6 and IL-8 production, and the expression of vascular endothelial growth factor (VEGF), monocyte chemoattractant protein-1 (MCP-1), and granulocyte macrophage colony-stimulating factor (GM-CSF), which promotes blood vessel growth and the recruitment of immune cells form the choroidal space, such as macrophages

Despite evidence for a molecular weight limit for the diffusion of proteins across BrM that decreases with age [77], little consideration has been given to the movement of complement regulators across BrM. However, recent diffusion studies with enriched BrM from human donor eyes over a wide age-range demonstrated that there is distinct selectivity in permeability of BrM to complement proteins with size and glycosylation being important determinants [78]. FHL-1, FD, and the anaphylatoxin C5a can diffuse freely, while the proteins including FI, FH, FB, and despite a lack of glycosylation, C3a cannot [78]. Therefore, complement proteins synthesized locally on either side of BrM, or on the choroidal side derived from the circulation, predominantly remain on their side of origin. This inevitably has consequences for the design of complement-targeting therapeutics as they may need to be designed to be delivered to specific sides of BrM at appropriate levels in order to confer the desired response without imbalancing complement homeostasis on the opposing side. It is interesting that RPE cells express C5aR, but not C3aR [79] and C5a, resulting from any complement activation in the choriocapillaris or intercapillary septa; could diffuse across BrM [78]; and illicit a response by the RPE to a perceived complement dysregulation (Fig. 2). If this is the case, targeting complement over-activation in the choriocapillaris may prevent the subsequent cascade of events that leads to C5a production, RPE cell stimulation, and secretion of various proteins that contribute to debris buildup and disruption of nutrient flow from the choroid to the RPE cell layer (Fig. 2). The size and glycosylation state of a therapeutic will likely dictate its passage through BrM and ability to modify complement activation on the opposite side to which it is initially delivered.

In some circumstances, the integrity of the BrM is compromised as evidenced by the invasion of macrophage infiltration into the macula [80] (perhaps recruited via prior complement activation) and subsequent filopodial projections through BrM into drusen [81]. Under these circumstances, the selectivity of the BrM is obviously going to be different. Similarly, the permeability of the outer blood-retinal barrier (comprising BrM and the RPE cell monolayer with tight junctions) will be altered in the eyes suffering late-stage geographic atrophy, where the local environment and tissue integrity are markedly different from (and unlikely to be translatable to) the early stages of disease. This latter point should also be considered when designing therapeutic interventions. Furthermore, the question remains as to whether regaining control of complement activation (driving early pathogenesis) *after* GA has begun will be sufficient to prevent the cascade of events leading to further cell death. It is possible that earlier-stage intervention to modulate complement will be a more effective approach in which case therapeutics delivered into the eye may need to be capable of crossing the intact outer blood-retinal barrier.

### Re-addressing complement over-activation in AMD

With regard to exploring complement-mediated therapies to treat ocular conditions, AMD currently represents the best and most timely example to use [82, 83]. Current knowledge suggests that AMD therapies should be directed towards regaining control of aberrant complement turnover locally in the macula, rather than focusing on systemic complement regulation. Therefore, there is a number of complement-mediated therapeutics already in use for other conditions that are currently being evaluated as treatments for AMD (see Table 1). With evidence suggesting that targeting the terminal pathway of complement is too late in the cascade to be effective in AMD, focus has been turning to regulating the amplification loop. C3 inhibitors, such as the cyclic peptide compstatin, have been used to moderate complement activation in other conditions [84] and have been proposed in AMD with a range of delivery methodologies. It remains to be seen what effects inhibiting the breakdown of C3 in the eye has given the need for the breakdown product iC3b to help induce immune tolerance in the eye [5]; it is of note that a phase II clinical trial investigating the C3 inhibitory compstatin analog AL-78898A in geographic atrophy patients (by intravitreal injection) had to be terminated (NCT01603043) because of adverse events. Recently, more promising trials have been conducted using intravitreal injections of lampalizumab, an anti-FD monoclonal antibody Fab fragment (which is now subject to two phase III clinical trials: NCT02247479 and NCT02247531) to treat geographic atrophy. It has been reported that an SNP associated with the *CFI* gene identified a subgroup of patients that responded best to this intervention. Given that FD is one of only a few soluble complement proteins that can diffuse through BrM and that IgG Fab domains can also pass through the BrM [78], it is uncertain where anatomically any therapeutic effect might be produced, with the retina, BrM, and the choriocapillaris all being possibilities. Current preclinical studies are also investigating anti-FB treatment (TA106) and intravitreal injections of FH [82].

**Table 1:** Current complement based therapeutics directed against AMD in clinical trials

Therapeutic (alt. name)^a^	Treatment type	Complement target	Company	AMD form targeted	Clinical trials
*AL-78898A* (*POT-4*)	Peptide	C3	Alcon	Dry	NCT01603043
					Phase II—terminated
*Zimura* (*ARC1905*)	Aptamer-based inhibitor	C5	Ophthotech	Dry	NCT00950638
					Phase I
				Wet	NCT00709527
					Phase I
*LFG316*	Monoclonal antibody		Novartis	Dry	NCT01527500
					Phase II
				Wet	NCT01535950
					Phase II
*CLG561* (*in combination with LFG316*)	Monoclonal antibody	Properdin	Alcon	Dry	NCT02515942
					Phase II
*Lampalizumab* (*FCD4514S*)	Antibody Fab fragment	Factor D	Genentech/Roche	Dry	NCT02247479
					NCT02247531
					Phase III
*AAVCAGsCD59*	Gene Therapy	CD59	Hemera Biosciences	Dry	NCT03144999
					Phase I

^a^In some instances, therapeutics have been known previously by a different name

One major factor hampering a successful complement-mediated therapy for AMD is mode of delivery. The failure of eculizumab to result in a slowing of the progression of GA in dry AMD patients [56] may have been due to any of a number of reasons including lack of stratification of patients based upon genetic risk, the trial intervened too late in the disease process, low drug dosage, or use of systemic rather than local delivery. Delivery of therapeutics directly into the eye could have advantages, but this does introduce new hurdles to overcome. For example, if intravitreal injection is used, the therapeutic needs to be able to reach the appropriate anatomical site, which might include the RPE, BrM, or choriocapillaris and achieve an effective concentration over a sustained period of time. The delivery of therapeutics by gene therapy, e.g., targeting the RPE, provides a potential solution as it would allow sustained delivery locally. However, it would be prudent to ensure that, if the therapeutic protein is basolaterally secreted, it does not become trapped at the RPE/BrM interface because of an inability to pass through BrM. Only the native soluble complement regulators FHL-1 and FD can diffuse through BrM given their small size and lack of glycosylation [78], whereas other larger complement regulators (e.g., FH or FI) may accumulate and have damaging effects. Diffusion through BrM is important if the therapeutic is to confer protection from complement dysregulation in BrM or the choriocapillaris.

## Conclusions

While the presence and activity of complement proteins in the eye has been known for many decades, the breadth and depth of complement involvement in immune homeostasis and immune tolerance in the eye is only recently becoming clear. As with many facets of biology, we learn the most when a system goes wrong. Complement turnover in the eye is fundamentally about a balance between controlled, slow, tick-over that promotes immune tolerance and stability, without losing the ability to respond when needed to defend host tissue and remove unwanted immune complexes. Any disruption to this fine balance can lead to dysregulation that contributes to inflammatory conditions of the eye. With regard to the exciting work currently ongoing in the field of AMD and new therapeutics, it is important that instead of diving headlong into novel therapeutic strategies, we take a moment to consider the unique anatomy of the human eye and give careful consideration to where complement over-activation should be addressed and at which stage of disease progression the therapy needs to be delivered.

## References

[1] Black JRM, Clark SJ (2016) Age-related macular degeneration: genome-wide association studies to translation. Genet Med. 10.1038/gim.2015.70 10.1038/gim.2015.70 PMC4823638 26020418

[2] McHarg S, Clark SJ, Day AJ, Bishop PN (2015) Age-related macular degeneration and the role of the complement system. Mol Immunol. 10.1016/j.molimm.2015.02.032 10.1016/j.molimm.2015.02.032 25804937

[3] Sohn JH, Kaplan HJ, Suk HJ (2000). Chronic low level complement activation within the eye is controlled by intraocular complement regulatory proteins. Investig Ophthalmol Vis Sci.

[4] Niederkorn JY (2007). The induction of anterior chamber-associated immune deviation. Chem Immunol Allergy.

[5] Sohn J-H, Bora PS, Suk H-J (2003). Tolerance is dependent on complement C3 fragment iC3b binding to antigen-presenting cells. Nat Med.

[6] Schmidt J, Klempp C, Büchler MW, Märten A (2006). Release of iC3b from apoptotic tumor cells induces tolerance by binding to immature dendritic cells in vitro and in vivo. Cancer Immunol Immunother.

[7] Bartel G, Brown K, Phillips R (2013). Donor specific transplant tolerance is dependent on complement receptors. Transpl Int.

[8] Mondino BJ, Chou HJ, Sumner HL (1996). Generation of complement membrane attack complex in normal human corneas. Investig Ophthalmol Vis Sci.

[9] Bora NS, Gobleman CL, Atkinson JP (1993). Differential expression of the complement regulatory proteins in the human eye. Invest Ophthalmol Vis Sci.

[10] Tang A, Marquart ME, Fratkin JD (2009). Properties of PASP: a pseudomonas protease capable of mediating corneal erosions. Investig Ophthalmol Vis Sci.

[11] Cocuzzi E, Guidubaldi J, Bardenstein DS (2000). Release of complement regulatory proteins from ocular surface cells in infections. Curr Eye Res.

[12] Tham YC, Li X, Wong TY (2014). Global prevalence of glaucoma and projections of glaucoma burden through 2040: a systematic review and meta-analysis. Ophthalmology.

[13] Shields MB (2008). Normal-tension glaucoma: is it different from primary open-angle glaucoma?. Curr Opin Ophthalmol.

[14] Miyahara T, Kikuchi T, Akimoto M (2003). Gene microarray analysis of experimental glaucomatous retina from cynomologous monkey. Investig Ophthalmol Vis Sci.

[15] Stasi K, Nagel D, Yang X (2006). Complement component 1Q (C1Q) upregulation in retina of murine, primate, and human glaucomatous eyes. Investig Ophthalmol Vis Sci.

[16] Kuehn MH, Kim CY, Ostojic J (2006). Retinal synthesis and deposition of complement components induced by ocular hypertension. Exp Eye Res.

[17] Williams PA, Tribble JR, Pepper KW (2016). Inhibition of the classical pathway of the complement cascade prevents early dendritic and synaptic degeneration in glaucoma. Mol Neurodegener.

[18] Yau JWY, Rogers SL, Kawasaki R (2012). Global prevalence and major risk factors of diabetic retinopathy. Diabetes Care.

[19] Zhang J, Gerhardinger C, Lorenzi M (2002). Early complement activation and decreased levels of glycosylphosphatidylinositol-anchored complement inhibitors in human and experimental diabetic retinopathy. Diabetes.

[20] Xu D, Yi H, Yu S (2016). Association of complement C5 gene polymorphisms with proliferative diabetic retinopathy of type 2 diabetes in a Chinese Han population. PLoS One.

[21] Davies CS, Harris CL, Morgan BP (2005). Glycation of CD59 impairs complement regulation on erythrocytes from diabetic subjects. Immunology.

[22] Ghosh P, Sahoo R, Vaidya A (2015). Role of complement and complement regulatory proteins in the complications of diabetes. Endocr Rev.

[23] Giles JL, Choy E, van den Berg C (2015). Functional analysis of a complement polymorphism (rs17611) associated with rheumatoid arthritis. J Immunol.

[24] Yang MM, Wang J, Ren H (2016). Genetic investigation of complement pathway genes in type 2 diabetic retinopathy: an inflammatory perspective. Mediat Inflamm.

[25] Caspi RR (2006). Mechanisms underlying autoimmune uveitis. Drug Discov Today Dis Mech.

[26] Jha P, Sohn J-H, Xu Q (2006). The complement system plays a critical role in the development of experimental autoimmune anterior uveitis. Invest Ophthalmol Vis Sci.

[27] Jha P, Sohn J-H, Xu Q (2006). Suppression of complement regulatory proteins (CRPs) exacerbates experimental autoimmune anterior uveitis (EAAU). J Immunol.

[28] Wong WL, Su X, Li X (2014). Global prevalence of age-related macular degeneration and disease burden projection for 2020 and 2040: a systematic review and meta-analysis. Lancet Glob Health.

[29] Whitmore SS, Sohn EH, Chirco KR (2015). Complement activation and choriocapillaris loss in early AMD: implications for pathophysiology and therapy. Prog Retin Eye Res.

[30] Mullins RF, Schoo DP, Sohn EH (2014). The membrane attack complex in aging human choriocapillaris. Am J Pathol.

[31] Keenan TDL, Toso M, Pappas C (2015). Assessment of proteins associated with complement activation and inflammation in maculae of human donors homozygous risk at chromosome 1 CFH-to-F13B. Investig Ophthalmol Vis Sci.

[32] Booij JC, Baas DC, Beisekeeva J (2010). The dynamic nature of Bruch’s membrane. Prog Retin Eye Res.

[33] Anderson DH, Radeke MJ, Gallo NB (2009). The pivotal role of the complement system in aging and age-related macular degeneration: hypothesis re-visited. Prog Retin Eye Res.

[34] Whitcup SM, Sodhi A, Atkinson JP (2013). The role of the immune response in age-related macular degeneration. Int J Inflamm.

[35] Schramm EC, Clark SJ, Triebwasser MP et al (2014) Genetic variants in the complement system predisposing to age-related macular degeneration: a review. Mol Immunol 61:118–125. 10.1016/j.molimm.2014.06.032 10.1016/j.molimm.2014.06.032 PMC4149817 25034031

[36] Fritsche LG, Igl W, Bailey JNC (2016). A large genome-wide association study of age-related macular degeneration highlights contributions of rare and common variants. Nat Genet.

[37] Sofat R, Casas JP, Webster AR (2012). Complement factor h genetic variant and age-related macular degeneration: effect size, modifiers and relationship to disease subtype. Int J Epidemiol.

[38] Fritsche LG, Chen W, Schu M (2013). Seven new loci associated with age-related macular degeneration. Nat Genet.

[39] Seddon JM, Yu Y, Miller EC (2013). Rare variants in CFI, C3 and C9 are associated with high risk of advanced age-related macular degeneration. Nat Genet.

[40] Kavanagh D, Yu Y, Schramm EC (2015). Rare genetic variants in the CFI gene are associated with advanced age-related macular degeneration and commonly result in reduced serum factor I levels. Hum Mol Genet.

[41] Johnson LV, Leitner WP, Staples MK, Anderson DH (2001). Complement activation and inflammatory processes in Drusen formation and age related macular degeneration. Exp Eye Res.

[42] Morgan BP, Harris CL (2015). Complement, a target for therapy in inflammatory and degenerative diseases. Nat Rev Drug Discov.

[43] Ripoche J, Day AJ, Harris TJ, Sim RB (1988). The complete amino acid sequence of human complement factor H. Biochem J.

[44] Hageman GS, Anderson DH, Johnson LV (2005). A common haplotype in the complement regulatory gene factor H (HF1/CFH) predisposes individuals to age-related macular degeneration. Proc Natl Acad Sci U S A.

[45] Haines JL, Hauser MA, Schmidt S (2005). Complement factor H variant increases the risk of age-related macular degeneration. Science.

[46] Klein RJ, Zeiss C, Chew EY (2005). Complement factor H polymorphism in age-related macular degeneration. Science.

[47] Edwards AO, Ritter R, Abel KJ (2005). Complement factor H polymorphism and age-related macular degeneration. Science.

[48] Day AJ, Willis AC, Ripoche J, Sim RB (1988). Sequence polymorphism of human complement factor H. Immunogenetics.

[49] Sivaprasad S, Adewoyin T, Bailey TA (2007). Estimation of systemic complement C3 activity in age-related macular degeneration. Arch Ophthalmol.

[50] Scholl HPN, Issa PC, Walier M (2008). Systemic complement activation in age-related macular degeneration. PLoS One.

[51] Silva AS, Teixeira AG, Bavia L (2012). Plasma levels of complement proteins from the alternative pathway in patients with age-related macular degeneration are independent of complement factor H Tyr^402^His polymorphism. Mol Vis.

[52] Stanton CM, Yates JRW, den Hollander AI (2011). Complement factor D in age-related macular degeneration. Invest Ophthalmol Vis Sci.

[53] Smailhodzic D, Klaver CCW, Klevering BJ (2012). Risk alleles in CFH and ARMS2 are independently associated with systemic complement activation in age-related macular degeneration. OPHTHA.

[54] Reynolds R, Hartnett ME, Atkinson JP (2009). Plasma complement components and activation fragments: associations with age-related macular degeneration genotypes and phenotypes. Investig Ophthalmol Vis Sci.

[55] Mullins RF, Dewald AD, Streb LM (2011). Elevated membrane attack complex in human choroid with high risk complement factor H genotypes. Exp Eye Res.

[56] Yehoshua Z, de Amorim Garcia Filho CA, Nunes RP (2014). Systemic complement inhibition with eculizumab for geographic atrophy in age-related macular degeneration: the COMPLETE study. Ophthalmology.

[57] Ristau T, Paun C, Ersoy L (2014). Impact of the common genetic associations of age-related macular degeneration upon systemic complement component C3d levels. PLoS One.

[58] Hecker LA, Edwards AO, Ryu E (2009). Genetic control of the alternative pathway of complement in humans and age-related macular degeneration. Hum Mol Genet.

[59] Khandhadia S, Hakobyan S, Heng LZ (2013). Age-related macular degeneration and modification of systemic complement factor H production through liver transplantation. Ophthalmology.

[60] Johnson PT, Betts KE, Radeke MJ (2006). Individuals homozygous for the age-related macular degeneration risk-conferring variant of complement factor H have elevated levels of CRP in the choroid. Proc Natl Acad Sci U S A.

[61] Clark SJ, Schmidt CQ, White AM et al (2014) Identification of factor h-like protein 1 as the predominant complement regulator in Bruch’s membrane: implications for age-related macular degeneration. J Immunol. 10.4049/jimmunol.1401613 10.4049/jimmunol.1401613 PMC4225158 25305316

[62] Clark SJ, Perveen R, Hakobyan S (2010). Impaired binding of the age-related macular degeneration-associated complement factor H 402H allotype to Bruch’s membrane in human retina. J Biol Chem.

[63] Clark SJ, Keenan TDL, Fielder HL (2011). Mapping the differential distribution of glycosaminoglycans in the adult human retina, choroid, and sclera. Invest Ophthalmol Vis Sci.

[64] Clark SJ, Bishop PN, Day AJ (2010). Complement factor H and age-related macular degeneration: the role of glycosaminoglycan recognition in disease pathology. Biochem Soc Trans.

[65] Clark SJ, Ridge LA, Herbert AP (2013). Tissue-specific host recognition by complement factor H is mediated by differential activities of its glycosaminoglycan-binding regions. J Immunol.

[66] Langford-Smith A, Day AJ, Bishop PN, Clark SJ (2015) Complementing the sugar code: role of GAGs and sialic acid in complement regulation. Front Immunol. 10.3389/fimmu.2015.00025 10.3389/fimmu.2015.00025 PMC4313701 25699044

[67] Clark SJ, Bishop PN, Day AJ (2013). The proteoglycan glycomatrix: a sugar microenvironment essential for complement regulation. Front Immunol.

[68] Langford-Smith A, Keenan TDL, Clark SJ (2014). The role of complement in age-related macular degeneration: heparan sulphate, a ZIP code for complement factor H?. J Innate Immun.

[69] Keenan TDL, Pickford CE, Holley RJ (2014). Age-dependent changes in heparan sulfate in human Bruch’s membrane: implications for age-related macular degeneration. Invest Ophthalmol Vis Sci.

[70] Raychaudhuri S, Iartchouk O, Chin K (2011). A rare penetrant mutation in CFH confers high risk of age-related macular degeneration. Nat Genet.

[71] Sánchez-Corral P, Pérez-Caballero D, Huarte O (2002). Structural and functional characterization of factor H mutations associated with atypical hemolytic uremic syndrome. Am J Hum Genet.

[72] Bhutto IA, Baba T, Merges C (2011). C-reactive protein and complement factor H in aged human eyes and eyes with age-related macular degeneration. Br J Ophthalmol.

[73] Thiele JR, Habersberger J, Braig D (2014). Dissociation of pentameric to monomeric C-reactive protein localizes and aggravates inflammation: in vivo proof of a powerful proinflammatory mechanism and a new anti-inflammatory strategy. Circulation.

[74] Molins B, Fuentes-Prior P, Adán A (2016). Complement factor H binding of monomeric C-reactive protein downregulates proinflammatory activity and is impaired with at risk polymorphic CFH variants. Sci Rep.

[75] Sjöberg AP, Trouw LA, Clark SJ (2007). The factor H variant associated with age-related macular degeneration (His-384) and the non-disease-associated form bind differentially to C-reactive protein, fibromodulin, DNA, and necrotic cells. J Biol Chem.

[76] Bhutto I, Lutty G (2012). Understanding age-related macular degeneration (AMD): relationships between the photoreceptor/retinal pigment epithelium/Bruch’s membrane/choriocapillaris complex. Mol Asp Med.

[77] Moore DJ, Clover GM (2001). The effect of age on the macromolecular permeability of human Bruch’s membrane. Invest Ophthalmol Vis Sci.

[78] Clark SJ, McHarg S, Tilakaratna V, et al (2017) Permeability of Bruch's membrane to complement proteins: Impications for translational medicine in AMD. Mol Immunol 89:200. 10.1016/j.molimm.2017.06.225

[79] Skeie JM, Fingert JH, Russell SR (2010). Complement component C5a activates ICAM-1expression on human choroidal endothelial cells. Investig Ophthalmol Vis Sci.

[80] McLeod DS, Bhutto I, Edwards MM (2016). Distribution and quantification of choroidal macrophages in human eyes with age-related macular degeneration. Investig Ophthalmol Vis Sci.

[81] Hageman GS, Luthert PJ, Victor Chong NH (2001). An integrated hypothesis that considers drusen as biomarkers of immune-mediated processes at the RPE-Bruch’s membrane interface in aging and age-related macular degeneration. Prog Retin Eye Res.

[82] Cantsilieris S, Schache M, Ashdown ML, Baird PN (2009). Recent patents relating to diagnostic advances in age related macular degeneration (AMD). Recent Pat DNA Gene Seq.

[83] Sahebjada S, Cantsileris S, Baird PN (2011). Gene patents related to common diseases of the eye. Recent Pat DNA Gene Seq.

[84] Ricklin D, Lambris JD (2013). Complement in immune and inflammatory disorders: therapeutic interventions. J Immunol.

